# Account of Diseases in an Eastern District of London, from the 20th of January, to the 20th of February, 1799

**Published:** 1799-03

**Authors:** 


*Account of Diseases in an Eastern District of London, from the 20th of January, to the 20th of February*, 1799.

| | |
|---|---|
| ACUTE DISEASES. | |
| Typhus Mitior - - | 2 |
| Pleurisy - - - - - | 4 |
| Peripneumony - - - | 5 |
| Peripneumonia-notha - - | 12 |
| Small-pox - - - - : | 6 |
| Acute Rheumatism - - | 3 |
| CHRONIC DISEASES. | |
| Dyspnœa - - - - - | 15 |
| Cough - - - - - | 25 |
| Cough and Dyspnœa - - | 17 |
| Hoarseness - - - - - | 6 |
| Phthisis Pulmonalis - - | 4 |
| Hæmoptoe - - - - - | 3 |
| Ascites - - - - - | 1 |
| Hydrops Pectoris - - - | 3 |
| Anasarca - - - - - | 2 |
| Cephalalgia - - - | 5 |
| Vertigo - - - - - | 2 |
| Opthalmia - - - - - | 3 |
| Epistaxis - - - - - | 1 |
| Menorrhagia Difficilis - - | 3 |
| Amenorrhœa - - - | 3 |
| Chlorosis - - - - - | 4 |
| Fluor Albus - - - | 3 |
| Enterodynia - - - - | 2 |
| Diarrhœa - - - - - | 4 |
| Dyspepsia - - - - - | 6 |
| Obstipatio - - - | 3 |
| Gastrodynia - - - - | 2 |
| Colica Pictonum - - | 1 |
| Dysuria - - - - - | 3 |
| Hæmorrhois - - - - | 1 |
| Hernia - - - - - | 1 |
| Jaundice - - - - - | 2 |
| Erysipelas - - - - | 1 |
| Paralysis - - - - - | 2 |
| Hypochondriasis - - | 4 |
| Chronic Rheumatism - - | 20 |
| PUERPERAL DISEASES. | |
| Ephemera - - - | 3 |
| Peritonitis - - - - | 1 |
| Menorrhagia Lochialis - | 2 |
| INFANTILE DISEASES. | |
| Aphthæ - - - - - | 3 |
| Opthalmia - - - - | 2 |
| Herpetic Eruptions - - | 1 |
| Vermes - - - - - | 2 |

The severe degree, and long continuance of cold, has been productive of different diseases affecting the pulmonary system.

Coughs, dyspnœa, catarrh, and peripneumony, have prevailed in an uncommon degree. The last of these diseases, particularly that species of it which is called peripneumonia-notha has been severely felt by many persons in the decline of life; and to a great number it has proved fatal.

This disease sometimes approaches in a very insidious manner; and in those patients who have long been subject to catarrhal affections it does not excite much alarm, and on that account is neglected, till it assume a formidable aspect. In addition to the common symptoms of catarrh, frequently of a greater or less degree of permanent inflammation serves to characterise the disease.

A sense of weight about the præcordia, with a difficulty of breathing, first announces the approach of this disease; respiration is performed rather with *difficulty* than with *pain*; or if pain is occasioned, it is rather of the obtuse than of the acute kind. The cough at the beginning is dry and hard; but afterwards a quantity of mucus, of various colour and consistence, is thrown up. A continuance of this expectoration with an abatement of other symptoms forms a favourable prognosis, respecting the termination of the disease. The pulse is frequently weak and irregular. In some instances, considerable pain is is felt in the head; a bloated countenance, and livid appearance about the cheeks and lips, indicate a difficult return of blood from the head. In the early ſtage of this diſeaſe, antimonial preparations are more proper than thoſe expectorants which are more heating and ſtimulating; and when accompanied with mild diluting drink, they promote a gentle diaphoreſis. Bliſters often afford an alleviation of ſymptoms, eſpecially when pain in the ſide, or in any part of the cheſt, becomes urgent. In one of the patients referred to in the foregoing liſt, this ſymptom was ſo urgent as to render it neceſſary to take away a few ounces of blood, by which means, together with the application of a bliſter to the part affected, conſiderable relief was obtained. In advanced ſtages of the diſeaſe, the different preparations of ſquills, and ſometimes the lac ammoniacum, prove very uſeful expectorants, and promote a favourable termination of the diſeaſe.

